# Deep learning application for the classification of Alzheimer’s disease using ^18^F-flortaucipir (AV-1451) tau positron emission tomography

**DOI:** 10.1038/s41598-023-35389-w

**Published:** 2023-05-19

**Authors:** Sang Won Park, Na Young Yeo, Yeshin Kim, Gihwan Byeon, Jae-Won Jang

**Affiliations:** 1 Department of Neurology, Kangwon National University Hospital, 156, Baengnyeong-ro, Chuncheon, Gangwon Republic of Korea; 2 Department of Medical Informatics, Kangwon National University, Chuncheon, Republic of Korea; 3 Department of Big Data Medical Convergence, Kangwon National University, Chuncheon, Republic of Korea; 4 School of Medicine, Kangwon National University, Chuncheon, Republic of Korea; 5 Department of Psychiatry, Kangwon National University Hospital, Chuncheon, Republic of Korea

**Keywords:** Neurology, Alzheimer's disease

## Abstract

The positron emission tomography (PET) with ^18^F-flortaucipir can distinguish individuals with mild cognitive impairment (MCI) and Alzheimer’s disease (AD) from cognitively unimpaired (CU) individuals. This study aimed to evaluate the utility of ^18^F-flortaucipir-PET images and multimodal data integration in the differentiation of CU from MCI or AD through DL. We used cross-sectional data (^18^F-flortaucipir-PET images, demographic and neuropsychological score) from the ADNI. All data for subjects (138 CU, 75 MCI, 63 AD) were acquired at baseline. The 2D convolutional neural network (CNN)-long short-term memory (LSTM) and 3D CNN were conducted. Multimodal learning was conducted by adding the clinical data with imaging data. Transfer learning was performed for classification between CU and MCI. The AUC for AD classification from CU was 0.964 and 0.947 in 2D CNN-LSTM and multimodal learning. The AUC of 3D CNN showed 0.947, and 0.976 in multimodal learning. The AUC for MCI classification from CU had 0.840 and 0.923 in 2D CNN-LSTM and multimodal learning. The AUC of 3D CNN showed 0.845, and 0.850 in multimodal learning. The ^18^F-flortaucipir PET is effective for the classification of AD stage. Furthermore, the effect of combination images with clinical data increased the performance of AD classification.

## Introduction

Alzheimer’s dementia (AD) is the most common type of dementia among older adults^1,2^. In general, the progression of AD can be divided into three stages: cognitively unimpaired (CU), mild cognitive impairment (MCI), and AD. Although AD is characterized by the pathological hallmarks of β amyloid (Aβ) deposition and tau neurofibrillary tangles (NFTs), tau burden is known to be more strongly associated with cognitive dysfunction than Aβ accumulation^3,4^. Accordingly, imaging of Aβ deposition, pathologic tau, and neurodegeneration forms a research framework [AT(N)]. Imaging of AD biomarkers is accomplished using amyloid positron emission tomography (PET) for Aβ, tau-PET for NFTs, and ^18^F-fluoro-deoxyglucose (FDG) PET or magnetic resonance imaging (MRI) for neurodegeneration^5^. These imaging techniques can be utilized to classify and stage AD^6,7^. For example, MRI and FDG-PET can reflect neurodegeneration, and amyloid PET can provide pathological evidence of Aβ agglomeration^8–10^. However, MRI and FDG-PET cannot specifically reflect the molecular pathological hallmarks of AD such as Aβ or tau burden. In addition, amyloid PET could be accumulated 20 years before the diagnosis of AD. It means that it is difficult to visualize the progression of Aβ accumulation because it is saturated at the time of disease^5^. On the contrary, tau-PET scans can directly reflect the pathological changes of AD and have a high correlation with cognitive function and disease progression^11^. In addition, cerebral structural changes also reveal a close relationship with pathological tau deposition^7,12^. The strength of tau-PET images is their ability to reveal tau accumulation patterns and specific deposit sites in focal regions of the brain similar to that shown by tau histology performed through Braak staging^7,13^.

In response to the vast increase in the amount of medical imaging data, deep learning (DL) of medical images have been used for disease classification. Although many studies have used DL for classification of AD, they have mainly focused on MRI, amyloid PET, or co-registration of both types of images^14–16^. In addition, many previous studies on DL application focused on models using AD images limited to specific parts of the brain related to cognitive function^17–19^. Application of a convolutional neural network (CNN) to tau-PET scans is a novel approach, as the spatial characteristics and interpretation of this modality are quite different than amyloid PET, FDG-PET, or MRI. In particular, the PET signal highlights the specific region of tau molecular manifestation in the brain and is considered more informative than other imaging techniques. This can have implications for CNNs, which require processing of complex inputs as well as visualization of informative features.

In this study, we implemented a DL framework for the classification of AD stage using ^18^F-flortaucipir PET. Transfer learning (TL) for high classification performance was performed using the weight derived from CU versus AD classification for CU versus MCI classification. By identifying the phenotype of tau deposition through two-dimensional (2D) and three-dimensional (3D) ^18^F-flortaucipir-PET molecular imaging based on DL, the clinical usefulness of ^18^F-flortaucipir-PET is proposed.

## Results

### Subject characteristics

The characteristics of all subjects investigated in this study are presented in Table 1. The mean age was 71.4 years, with 70.0 years in the CU, 72.0 years in the MCI, and 73.7 years in the AD groups. One hundred thirty-six (49.3%) were female and 140 (50.7%) were male, with 47 (62.7%) and 40 (63.5%) males present in the MCI and AD groups, respectively. As a result of the normality test, all covariates had p > 0.05. The differences among the three groups for all variables showed p < 0.05 as a result of one-way ANOVA. The total Aβ positive was 141, with 43 of CU (22 of florbetapir and 21 of florbetaben), 46 of MCI (18 of florbetapir and 28 of florbetaben), and 52 of AD (23 of florbetapir and 29 of florbetaben).

**Table 1:** Subject characteristics.

	Total (N = 276)	CU (N = 138)	MCI (N = 75)	AD (N = 63)
Age*	71.4 ± 7.1	70.0 ± 5.8	72.0 ± 7.8	73.7 ± 8.2
Sex**				
Female	136 (49.3%)	85 (61.6%)	28 (38.4%)	23 (38.5%)
Male	140 (50.7%)	53 (38.4%)	45 (61.6%)	40 (61.5%)
Education (years)*	16.4 ± 2.3	16.9 ± 2.2	16.5 ± 2.2	15.4 ± 2.5
MMSE score**	27.2 ± 3.1	29.0 ± 1.1	27.3 ± 2.4	22.9 ± 2.7
Amyloid (−/ +)	135/141	95/43	29/46	11/52
^18^F-Florbetapir (−/ +)	53/63	42/22	7/18	4/23
^18^F-Florbetaben (−/ +)	82/78	53/21	22/28	7/29

The superscript * means that the variable has statistical significance(p < 0.05) and ** means that the variable has significance(p < 0.001) among the three groups by one-way ANOVA. Values are presented as mean ± SD unless otherwise stated.

*CU* cognitive unimpaired, *MCI* mild cognitive impairment, *AD* Alzheimer’s disease, *MMSE* Mini-Mental State Examination.

### Classification performance between CU and AD

The CU and AD classification results are shown in Table 2. Most of the result metrics in the 2D CNN-LSTM and 3D CNN models showed that the multimodal performance was slightly more significant compared to the image classification. For the 2D multimodal results, the receiver operating characteristic (ROC) area under the curve (AUC) was 0.947, accuracy 88.5%, precision 86.7%, recall 92.9%, F1 score 89.7% and specificity 84.6%. The 3D multimodal results were higher than those of image classification in all performance indicators, with AUC of 0.976, accuracy 92.3%, precision 92.9%, recall 92.9%, F1 score 92.9% and specificity 92.3% suggesting better performance than the 2D model.

**Table 2:** Results of classification by deep learning using 18F-flortaucipir-PET.

Classification	Dimension	Type	AUC	Accuracy	Precision	Recall	F1 score	Specificity
	2D	Image	0.964	0.885	0.857	0.923	0.889	0.846
		Multimodal	0.947	0.889	0.867	0.929	0.897	0.846
	3D	Image	0.947	0.885	0.917	0.846	0.880	0.923
		Multimodal	0.976	0.923	0.929	0.929	0.929	0.917
CU-MCI	2D	Image	0.840	0.800	0.750	0.857	0.800	0.750
		Multimodal	0.923	0.833	0.786	0.846	0.815	0.824
	3D	Image	0.845	0.833	0.824	0.875	0.848	0.786
		Multimodal	0.850	0.828	0.867	0.813	0.839	0.846

The multimodal images consist of features extracted from PET images through CNN models and clinical variables such as age, sex, education, and MMSE.

*AUC* area under the receiver operating characteristic curve, *CU* cognitively unimpaired, *MCI* mild cognitive impairment, *AD* Alzheimer’s disease, *PET* positron emission tomography.

### Classification performance in between CU and MCI

**Figure 1:**
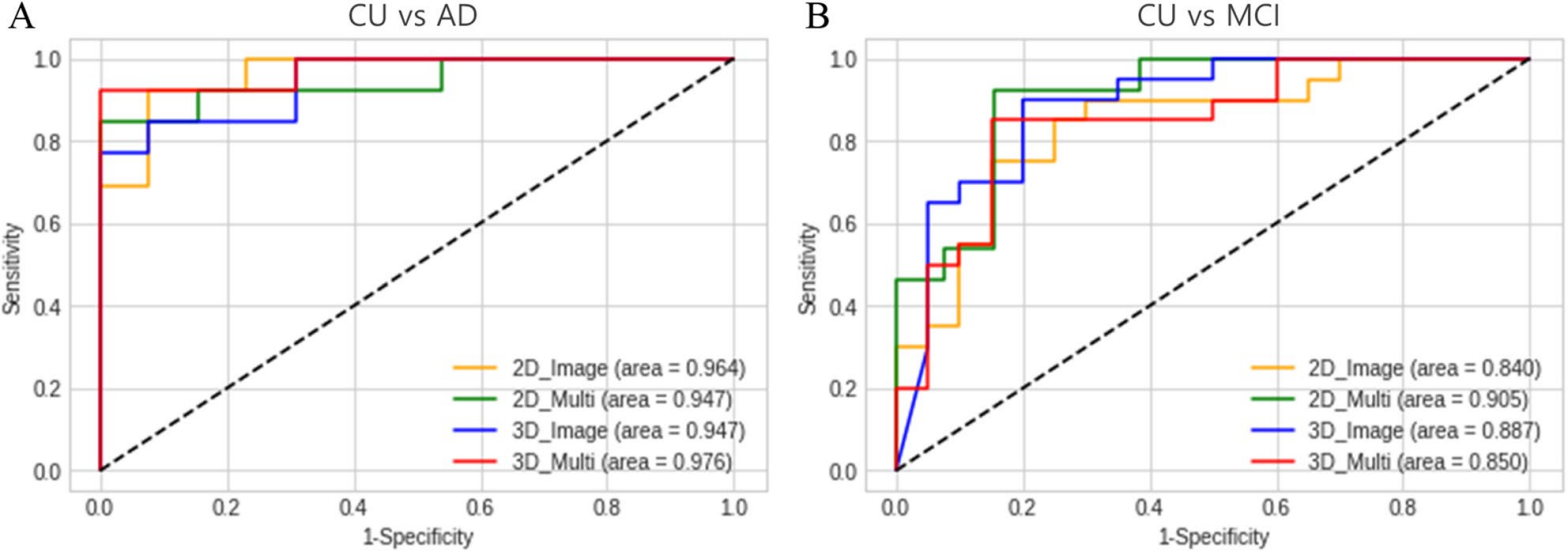
The receiver operating characteristic curve of classification results between (**A**) CU and AD (**B**) CU and MCI. *AD* Alzheimer’s disease, *CU* cognitively unimpaired, *MCI* mild cognitive impairment.

The CU and MCI classification results are shown in Table 2. The multimodal performance was slightly higher than image classification result metrics of 2D CNN-LSTM and 3D CNN. The 2D multimodal results were the same or higher than those of image classification, with AUC of 0.923, accuracy 83.3%, precision 78.6%, recall 84.6%, F1 score 81.5% and specificity 82.4%. The 3D multimodal results show AUC of 0.850, and accuracy 82.8%, precision 86.7%, recall 81.3%, F1 score 83.9% and specificity 84.6%, which were higher than those of image classification in all performance indicators (Fig. 1).

### Identification of informative features for AD classification

**Figure 2:**
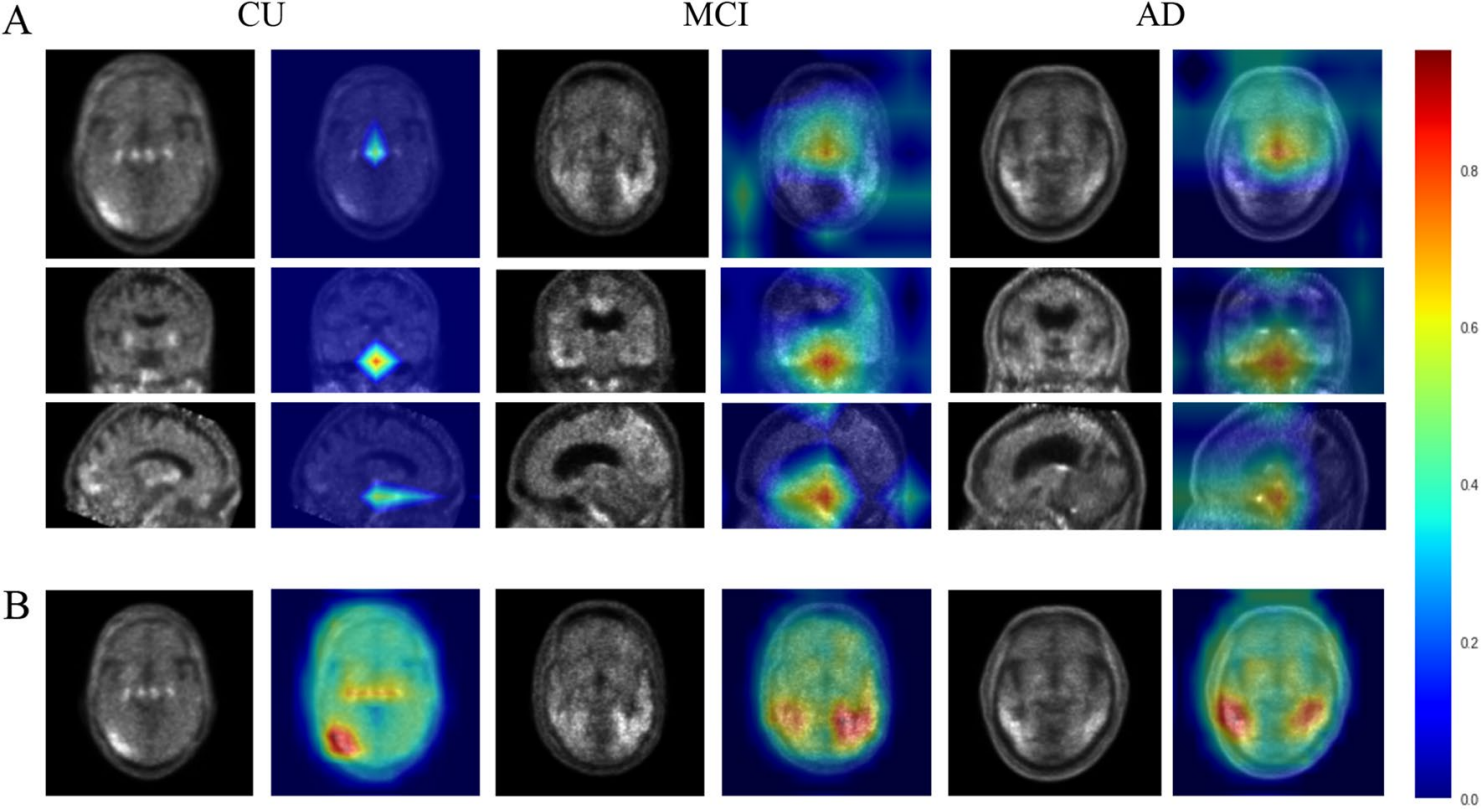
The results presented by GRAD-CAM for (**A**) 3D CNN and (**B**) 2D CNN-LSTM. Color bar means that "blue-red" color schema as the min–max mapping of the values.

GRAD-CAM findings confirmed that 2D and 3D CNN learned through feature extraction from most areas in the image (Fig. 2). Figure 2A is the result of 3D CNN, and Fig. 2B is the result of 2D CNN-LSTM. The identification of informative features in the Grad-Cam results, the distinctive area extracted from the brain was an area associated with cognitive functions such as the hippocampus and the lateral and middle temporal regions. In addition, through 3D sagittal phase in AD group, it was able to observe some cingulate regions were included. As a result of 2D CNN-LSTM, the regions that appeared through GRAD-CAM via a single axial phase shows a lot of dependent parts of uptake region.

## Discussion

In this study, DL was used to grading and differentiate syndromal cognitive stage between CU and AD, CU and MCI. In the MCI and AD groups used in this study, it was confirmed that there were some subjects who showed amyloid and flortaucipir positive or amyloid and flortaucipir negative at the same time. This suggests that this study was performed the syndromal cognitive stage grading of MCI and AD through flortaucipir PET. The classification among CU, MCI, and AD performed in this study is a syndromic cognitive stage grading, and AI modeling based on DL were performed with the goal of grading between CU and MCI, and between CU and AD. In addition, by applying the Tau PET image -based DL technique, the possibility of clinical syndromal grading was presented through CU VS MCI and CU VS AD comparison. It means that it is significance as a preliminary study to create a numeric staging model^5^. The results of 2D CNN-LSTM and 3D CNN proved the high performance of the classification ability of these imaging biomarkers. Moreover, multimodal data integration was performed by adding the demographic and neuropsychological variables into the CNN models as a method to use quantitative data which could be acquired at screening or baseline for disease classification. In 2D CNN-LSTM image classification for distinguishing between CU and AD has an AUC of 0.964 and accuracy of 88.5%. In addition, the results of 3D CNN image classification showed the AUC of 0.947 and accuracy of 88.5%. In multimodal classification, the results of 2D CNN-LSTM and 3D CNN showed the AUC of 0.947 and 0.976, respectively. For distinguishing between CU and MCI in image classification task has an AUC of 0.840 and accuracy of 80.0%. In addition, the results of 3D CNN image classification showed the AUC of 0.845 and accuracy of 83.3%. In multimodal classification, the results of 2D CNN-LSTM and 3D CNN showed the AUC of 0.923 and 0.850, respectively.

This study has several novel features. First, the classifiers generated in this study demonstrated that accumulated tau tangles may have an important role in AD pathogenesis based on the characteristics of their distribution. Previous studies using an ADNI database-driven approach have determined that the principal regions of tau pathology mainly overlap with the Braak stage III regions of interest (ROIs) (i.e., the amygdala, para-hippocampal gyrus, and spindle)^6,17,20^. It is generally known that stage III/IV ROIs could be observed in patients with CU as well as those with AD, whereas stage I/II is common in patients with CU and stage V/VI is common in those with AD^21^. In other words, it is difficult to classify tau deposition measurements as representative of cognitive decline including MCI and AD compared to CU through the ROIs of stage III/IV. We performed a systematic review of the existing literature to summarize the most common CU versus AD classification techniques that include comparison of CU versus MCI (Table 3)^17,18,22–27^. Notably, the classification between CU and MCI in this study showed better performance than other previously published methods. Significant accuracy was achieved for distinguishing both classifications based on regions with accumulated tau, which were set in the DL models. In addition, we generated regions with important identified features by GRAD-CAM in the DL process. The left and right amygdala, and left entorhinal, left para-hippocampal, inferior temporal, and right middle-temporal regions were identified as the main tau deposition regions. This suggests that tau deposition in the regions revealed by DL frameworks is similar to the regions of neurodegenerative and cognitive decline identified by Braak staging. Moreover, by including the entorhinal and inferior-temporal regions, which are known to be affected in early AD, among the Braak stage I/II regions and suggesting their importance, the classifiers generated in this study reflect the tau accumulation characteristics of AD and reinforce the suggestion of previous studies regarding their important role in early pathogenesis^28,29^. The results of our study also correspond well with the tau pattern and related regions as reported in previous studies ^30–32^. Second, we conducted TL by applying the weights of the classifier between CU and AD for maximized performance of classification between CU and MCI. The result for classification CU and MCI in this study was able to provide better performance than other previously published methods (Table 3). In particular, by presenting the results of CU and MCI classification with higher performance than other studies, we present the possibility of syndromal cognitive staging in early stages, which has recently attracted attention. In addition, it was confirmed that the DL based classification performance (2D; AUC of 0.840 and 0.923, 3D; AUC of 0.845 and 0.850) is superior to existing conventional ML model performance (Tau SUVR; AUC of 0.720, Tau SUVR with clinical variables; AUC of 0.800) of support vector machine (SVM) based classification and effective for classifying grade staging between CU and MCI which are relatively difficult to distinguish (Table 2). In the classification between CU and AD through ML, the difference in continuous numeric variables such as MMSE and Tau SUVR is stark, and it can be shown that the effect is better than that of DL (Supplementary Table S1). However, for the classification of CU vs MCI, which is relatively difficult to distinguish in terms of clinical symptoms, the DL-based classification performance was superior to staging. This suggests the possibility of clinically useful use through future research development. Although there might be some differences in the model structure and method of feature extraction, our results suggest that good performance of the classification between CU and MCI is presented through the application of weights of classification between CU and AD within the same data set. Third, the classifiers of this study could be applied to measurements that are easily obtained in clinical practice. In this study, we trained the 2D model using consecutive 2D slices by stacking two consecutive LSTM. Of a total of 144 slices, the model in this study used 72 consecutive even-numbered slices. In many clinical applications, brain PET scans for AD require fewer slices than the number of slices used in this study with 2-mm or 3-mm axial 2D slice thickness. The results of this study indicate that there is a possibility to learn all data at once without omitting the specific axial image information of each individual patient. In addition, multimodal layering was performed by concatenation of demographic and neuropsychological variables with the flattened layer of features extracted through CNN before entering the LSTM. The combined clinical variables used in this study were age, sex, education, and MMSE score, which are easily obtainable indicators at the screening stage for AD clinical trials or in hospital visits of outpatients. Through the results of our multimodal models, we demonstrated that the combination of clinical information with images could help to improve model performance slightly more than that of image DL.

**Table 3:** The systematic review and comparison of classification by DL using PET.

	Modality	Model	Participant counts			Classification results (AUC)	
			CU	MCI	AD	CU-AD	CU-MCI
Choi and Jin (2018)	PET^b^	3D CNN	182	171	139	96.0	84.2
Li et al. (2015)	PET^b^	3D CNN	198	403 (167 of LMCI)	198	88.7	69.5
Wen et al. (2021)	PET^b^	3D CNN	835		490	82.1^d^	
		2D CNN				79.2^d^	
Lu et al. (2018)	PET^a^	DNN	378	626 (217 of LMCI)	238	84.5	85.9
Liu et al. (2018)	PET^a^	3D CNN	100	146	93	93.5	82.1
		2D CNN				91.8	78.8
Zou et al. (2021)	PET^c^	3D CNN	319	127			85.8^d^
		2D CNN					88.4^d^
Jo T et al. (2020)	PET^c^	3D CNN	66	168 (71 of LMCI)	66	90.4^d^	
This study	PET^c^	3D CNN	138	73	65	94.7	84.5
		Multimodal				97.6	85.0
		2D CNN				96.4	84.0
		Multimodal				94.7	92.3

*PET* positron emission tomography, *CNN* convolutional neural network, *AUC* area under curve, *CU* cognitive un-impairment, *MCI* mild cognitive impairment, *AD* Alzheimer’s disease, *DNN* deep neural network.

^a^FDG-PET.

^b^Amyloid PET.

^c18^F-flortaucipir-PET.

^d^Accuracy (%).

However, there were some limitations in this study. First, we conducted 2D CNN-LSTM modeling utilized only consecutive even-numbered 72 slices in the axial direction among total of 144 consecutive slices of 3D PET data. The selected contiguous 72 slices were acquired after resampling the data from initial ADNI (96 slices per patient, 1.2 × 1.2 × 1.2 mm) to a voxel size of 1 × 1 × 1 mm. The method using consecutive even-numbered 72 slices was chosen as a way to overcome hardware limitations while maximally covering the entire brain volume area. As a result, we could present higher performance than other existing studies. However, these methods cannot be explained to completely cover the entire volume area, and some brain information is expected to be lost. If the hardware limitation is overcome in the future, the study could be conducted using total of 144 slices in the same process. Second, the small number of subjects was a problem. The data available in this study was less than that required for general DL because the ADNI 3 protocol was limited to 63 participants with AD. In DL training, if more samples are generally applied to the models, the better the results. Due to the small number of subjects, we allocated 20% of the training set for each cross-validation data set for validation. In addition, in the case of MCI subjects used in this study, as a late MCI, there was a limit for specific classification of syndromal cognitive staging with AD. In the future, when additional data is obtained using the model implemented in this study, it is possible to accurately grading for AD staging. In addition, the TL is used for applying a small data set through pre-trained models constructed from large data sets to obtain results with fine tuning. However, in this study, it was not possible to acquire many subjects; thus, the frozen layers method with feature extraction and cross-validation was performed to solve this problem and improve the reliability of the CU versus MCI classification model. Third, we use imperfect clinical diagnosis as the gold standard for modeling. As shown in Table 4, the clinical diagnosis presented in ADNI that we used is based on relatively objective criteria as a result of considering MMSE, CDR, logical memory test, and general cognition and function. In addition, it is being quality controlled by the ADNI clinical core, suggesting that many efforts are being made to compensate for incompleteness^33^. However, we need to conduct research using objective golden standards such as brain pathology or quantitative measures of biomarkers through study in the future. Lastly, the identification of extracted informational features for AD classification through GRAD-CAM shows a mixture of on-target binding and off-target binding. In particular, right off target binding is shown in the sagittal phase as a result of 3D CNN. This is seen as a limitation of flortaucirpir ligand, and effective research improvement can be presented through the second-generation tau ligand in the future. In addition, segmentation such as cortex, central structures and superior cerelleum before processing could be an alternative solution. In this study, we suggested that ^18^F-flortaucipir PET images could be a scalable biomarker by applying a DL framework for classification of AD stage. Our results show that the DL models using images in combination with clinical variables can effectively classify AD stages.

**Table 4:** Classification of ADNI to distinguish CU, MCI and dementia**.**

	CU	MCI	AD
Subjective memory complaint	None	Yes	Yes
MMSE score	≥ 24	≥ 24	Between 20 and 24 (exceptions for 24 and 25 for participants with less than 8 years of education)
CDR	CDR = 0 Memory box score must be 0	CDR = 0.5 Memory box score of at least 0.5	CDR = 0.5 or 1.0
Logical memory score	≥ 9 for 16 or more years of education ≥ 5 for 8–15 years of education ≥ 3 for 0–7 years of education	≤ 8 for 16 or more years of education ≤ 4 for 8–15 years of education ≤ 2 for 0–7 years of education	≤ 8 for 16 or more years of education ≤ 4 for 8–15 years of education ≤ 2 for 0–7 years of education
General cognition and functional status	Cognitively normal based on the absence of significant impairment in cognitive functions or activities of daily living	General cognition and functional performance sufficiently preserved such that a diagnosis of dementia cannot be made	NINCDS/ADRDA criteria for probable AD

This table was adapted and modified from the procedure manuals for ADNI3 available at http://adni.loni.usc.edu/methods/documents/.

*ADNI* Alzheimer’s Disease Neuroimaging Initiative, *CU* cognitively unimpaired, *MCI* mild cognitive impairment, *AD* Alzheimer’s disease, *MMSE* Mini-Mental State Examination, *CDR* The Clinical Dementia Rating Scale, *NINCDS/ADRDA* National Institute of Neurological and Communication Disorders and Stroke/Alzheimer’s Disease and Related Disorders Association.

## Methods

### Subjects

**Figure 3:**
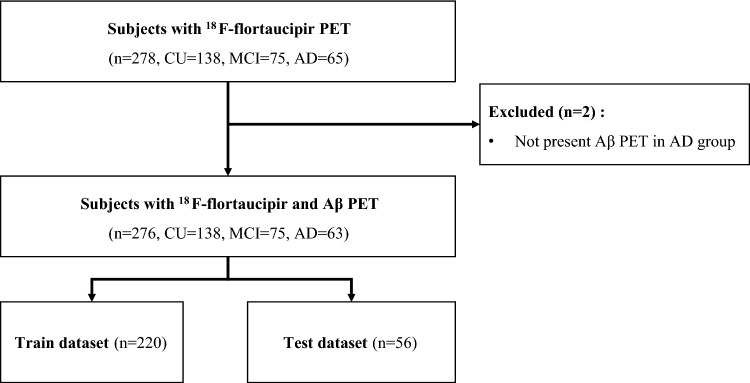
Subjects flowchart through this study for the training and test datasets. Within the training data set, 20% was used as validation data and cross-validation was performed 5 times. Within the ADNI3 data set, 'AV1451 Coreg, Average, Standardized Image and Voxel Size' PET images were used, and each group (CU, MCI, AD) was classified based on clinical syndrome staging by registered in ADNI cohort. *Aβ* β-amyloid, *PET* positron emission tomography.

A total of 271 subjects (138 CU, 75 MCI, and 63 AD) in the Alzheimer’s Disease Neuroimaging Initiative (ADNI3) for whom ^18^F-flortaucipir PET scans were performed at baseline were recruited. Age, sex, education, Mini-Mental State Examination (MMSE) score, ^18^F-flortaucipir-PET images, and diagnostic results were acquired (Fig. 3). All subjects were divided using criteria provided as clinical syndrome diagnoses within the ADNI cohort (Supplementary Table S2, S3). All subjects in the CU group had clinical dementia rating (CDR) scores of 0 or 0.5, which allowed them to be distinguished from participants with MCI and AD. The patients with MCI did not meet the dementia criteria and were evaluated based on an objective memory impairment determination. All participants with MCI had MMSE scores of 24 or higher up to 30 and CDR scores of 0.5, a CDR memory score of 0.5 or higher. In addition, d a score that indicated impairment on the delayed recall of Story A of the Wechsler Memory Scale-Revised (≥ 16 years of education: < 11; 8–15 years of education: ≤ 9; 0–7 years of education: ≤ 6) was applied^34^. All patients that met the criteria for AD had CDR scores of 0.5 of 1 and a score that indicated impairment on the delayed recall of Story A of the Wechsler Memory Scale-Revised (≥ 16 years of education: ≤ 8; 8–15 years of education: ≤ 4; 0–7 years of education: ≤ 2). A final total of 271 subjects from the ADNI3 cohort were selected for this study (Table 4).

The study procedures were approved for all participating centers (https://adni.loni.usc.edu/wp-content/uploads/how_to_apply/ADNI_Acknowledgement_List.pdf), and written informed consent was obtained from all participants or their authorized representatives. A committee on human research at each participating institution approved the study protocol, and all participants or legal guardian(s)/legally authorized representatives gave their informed consent. In addition, all experiments were performed in accordance with the relevant guidelines and regulations outlined in the IRB.

### Data acquisition and preprocessing

^18^F-flortaucipir 3D dynamic PET scan images were acquired for all individuals. All PET images were acquired by a 30-min scan, 75–105 min after intravenous (IV) injection of ^18^F radio isotope (RI) with 370 mBq (10.0 mCi) ± 10% radioactivity, considering the weight of each patient, and flortaucipir ligand. For this study, pre-processed PET images (AV1451 Coreg, Average, Standardized Image, and Voxel Size) provided and described were acquired from the ADNI3 cohort. As all images were preprocessed such as anterior–posterior axis fitting to the anterior commissure-posterior commissure line. Scans were normalized to Montreal Neurologic Institute (MNI) space using parameters generated from segmentation of the T1-weighted MRI scan in Statistical Parametric Mapping v12 (SPM12).

Intensity normalization was performed using a cerebellar gray matter as a reference region and standard uptake value ratio (SUVR) could be acquired for RI uptake calculation for each region in the brain^35–37^. More details of ^18^F-flortaucipir-PET preprocessing can be found in other related studies^22,35,38^. After acquisition images we converted the voxel size to 1 × 1 × 1 mm by resampling and resizing and acquired 3D PET images to use input data for the development of the DL framework. For 2D CNN long short-term memory (LSTM) DL framework development, we extracted 72 even-numbered sequential axial slice images from a total of 144 3D images per individual subject. The 3D CNN DL framework was performed using total image. All data such as demographic and clinical information, image voxel size was processed for min–max normalization for a multimodal framework.

The data for both frameworks were split as 80% of the total data for the training set and 20% of the total data for the test set. The validation set was 20% of the training set. Five-fold cross-validation was applied to derive stable performance (Fig. 3). The data ratio was maintained during five cross-validations, as one subset was selected for testing and the remaining four sets for validation.

### Define of Aβ PET status

We downloaded the ^18^F-florbetapir and ^18^F-florbetaben analysis data from the ADNI. Moreover, we classified each participant as Aβ-positive PET scan on observing a global standardized uptake value ratio (SUVR) > 1.11 for the ^18^F-florbetapir ^39^. For ^18^F-florbetaben, tracer uptake was assessed according to the regional cortical tracer uptake system in four brain regions (frontal cortex, posterior cingulate cortex/precuneus, parietal cortex, and lateral temporal cortex) and the cut-off value was 1.1^40^.

### Classification for deep learning

TL was performed using the weight of both 2D CNN-LSTM and 3D CNN models built in classification between CU and AD to increase the classification between CU and MCI classification performance. In both 2D and 3D models, feature extraction methods similar to classification between CU and AD models was used by conducting a freezing technique to fix the feature extraction architecture for classification between CU and MCI by TL. From the first convolutional layer to the last layer (before the fully connected layer), which performs feature extraction within the image, it was frozen for TL, and DL was performed through a classifier composed of dense layers. The learning rate was changed to 0.00001, considering that it is more difficult to distinguish between CU and MCI. For each classification between CU and AD, CU and MCI, binary cross-entropy loss function was applied.

### 2D CNN-LSTM

**Figure 4:**
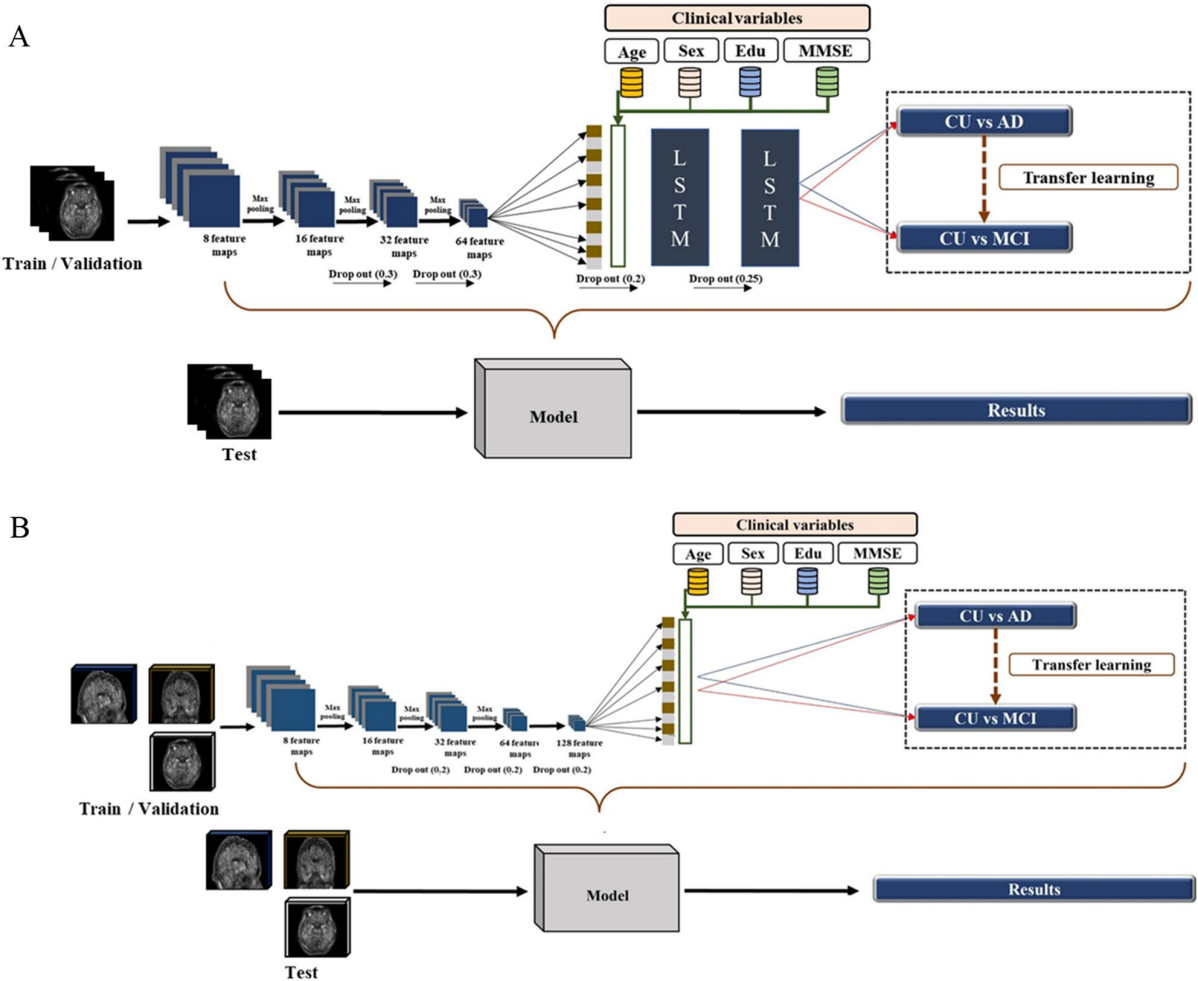
Deep learning framework for (**A**) 2D CNN-LSTM and (**B**) 3D CNN. *AD* Alzheimer’s disease, *CU* cognitively unimpaired, *MCI* mild cognitive impairment, *MMSE* Mini-Mental State Examination, *LSTM* long shot term memory.

**Figure 5:**
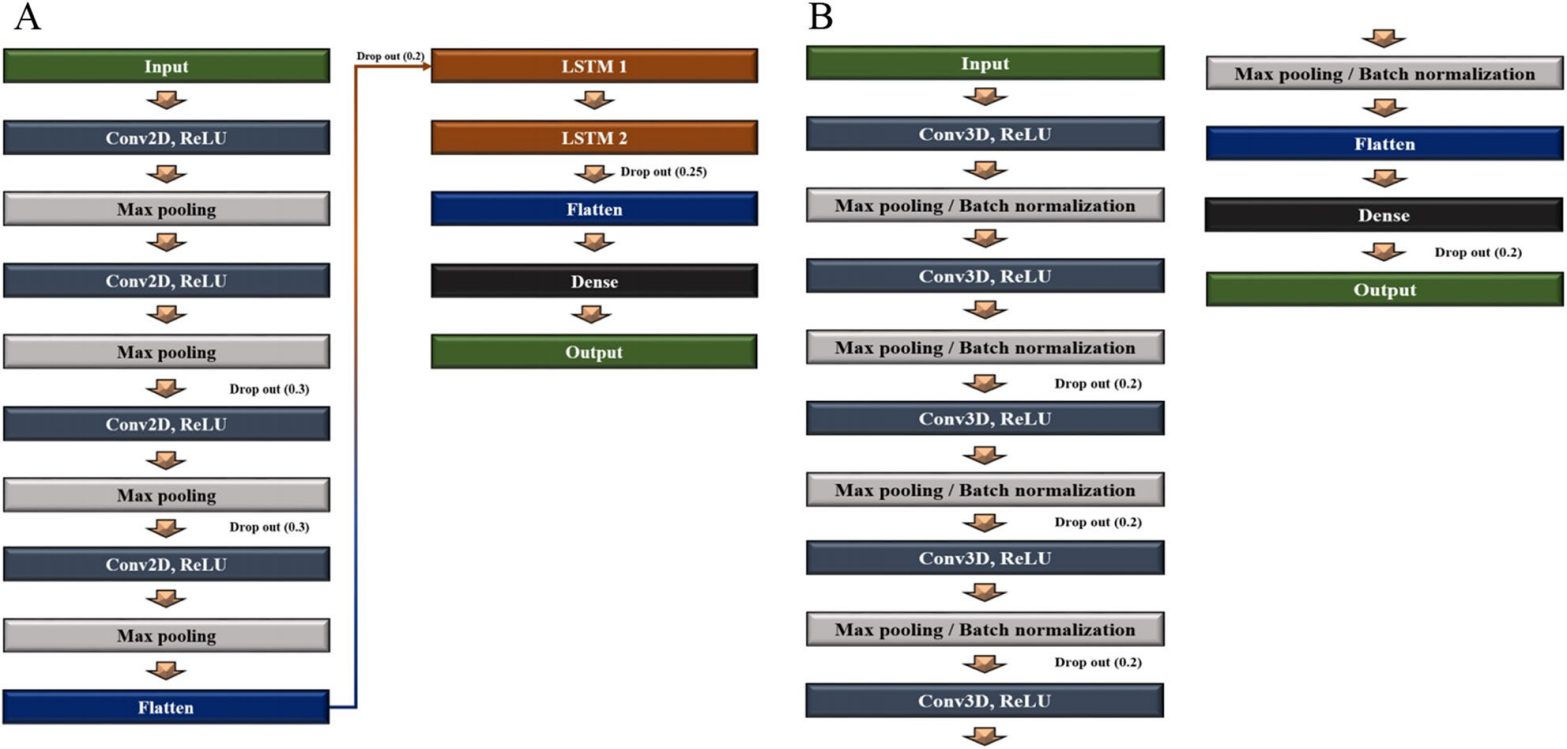
The process for layer stacking of deep learning model (**A**) 2D CNN-LSTM, (**B**) 3D CNN.

The 2D CNN model was prepared in conjunction with the LSTM (Fig. 4). Two LSTM algorithms were consecutively stacked after the 2D CNN to minimize the loss of brain information contained in the 72 axial images from the upper to the lower part of the head. All axial images were sequentially processed by LSTM configuration models after feature extraction from each slide through 2D CNN. Each slice index i and extracted features f were converted to the form of (*i*, *f*), and the model was constructed by stacking two LSTM layers consecutively. In the first layer of LSTM, the features of each slice are output in the form of (*i*, *f*) → (*i*, LSTM(output)). While maintaining the sequential slices information, the variable of *f*(Features) reduced by the size of the first LSTM output is input to the second LSTM layer, and finally output in the form of (*i*, LSTM(last output)). That is, the features corresponding to the entire slice information were sequentially extracted, and the model was constructed through two consecutive LSTM layers. To avoid excessive epochs that could lead to overfitting, early stopping was applied if the model did not show any improvement loss for ten iterations. The hyper-parameters for classification between CU and AD, adaptive moment estimation (Adam), a first-order gradient-based probability optimization algorithm with learning rate = 0.0001, decay rate = 0.96, and batch size of 1, was used (Fig. 5). The feature maps (8,16,32,64) were extracted from four hidden layers; kernel_size = 2, same padding, and Maxpool2D were applied to each layer to use the activation function of rectified linear unit (ReLU). Dropout (0.3) was applied to the third and fourth layers. Two LSTM (200,64) layers were applied, and dropout (0.25) was applied after the first layer.

### 3D CNN

The 3D CNN model was constructed more depth than 2D CNN-LSTM since the 3D images have volume including with height and width information (Fig. 4). To avoid overfitting, early stopping was applied if the model did not show any improvement loss for 15 iterations. Hyper-parameters for classification between CU and AD, such as optimization function, learning rate, decay rate, and batch size, were the same as for the 2D model. The feature maps (8,16,32,64,128) were extracted from four hidden layers; kernel_size = 3 and Maxpool3D were applied to each layer to use the activation function ReLU (Fig. 5). Features that had passed through the flattened layer were input into the three dense layers, and dropout (0.2) was subsequently applied.

### Informative feature identification for AD classification

Gradient-weighted class activation mapping (GRAD-CAM) was used to identify informative features extracted through CNN models. The feature map could be visualized with the average pixel value up to final layers. We identified regions in the brain as the ReLU activation function was applied to visualize important parts in the model during the analysis process.

### Evaluation performance

For the evaluation of the model performance, four metrics (accuracy, recall, precision, and F1 score) were used. Since this study focused on the accurate classification between CU and AD, CU and MCI, the metric of true positive was mainly established for overall performance evaluation of the classification model. The equations is Eqs. (1)–(4).

1 $$Accuracy= \frac{TP+TN}{TP+TN+FP+FN}$$

2 $$Precision= \frac{TP}{TP+FP}$$

3 $$Recall= \frac{TP}{TP+FN}$$

4 $$F1 \, Score= 2\times \frac{Precision\times Recall}{Precision+Recall}$$

### Statistical analysis

After performing the Shapiro–Wilk normality test on the variables from each group (CU, MCI, and AD), examination of significance differences among groups was conducted by one-way analysis of variance (ANOVA) and the chi-squared ($\upchi$^2^) test for continuous and categorical variables, respectively. After conducting Levene's test for checking the equality of variances, ANOVA was performed to test difference in the means among three groups. If the assumption of equal variance was not satisfied, Welch's ANOVA was performed to test the mean difference among groups. In addition, post-hoc analysis was performed using the Games-Howell test if the assumption of equal variance was established, and the Scheffe test if the assumption was not satisfied. Statistical significance was set at p < 0.05 and p < 0.001. All statistical analysis was performed in R (version 4.1.0).

### Tools

Tensorflow 2.8.0 and Keras 2.8.0 were used to construct DL frameworks and performed by scratch on Python 3.7.0 for all processing.

### Ethics approval and consent to participate

The study was approved by the institutional review boards of Kangwon National University Hospital (approval No. KNUH-2022-06-011) all participating institutions, and written informed consent was obtained from all participants or their authorized representatives.

## Supplementary Information

Supplementary Tables.

## Data Availability

All ADNI data used in this study is available through the ADNI website (https://adni.loni.usc.edu/data-samples/access-data/). The datasets used and/or analyzed during the current study available from the corresponding author on reasonable request.
