# Supplementary material for: Deep learning application for the classification of Alzheimer’s disease using 18F-flortaucipir (AV-1451) tau positron emission tomography
Source: Sci Rep. 2023 May 19;13:8096. doi: 10.1038/s41598-023-35389-w (PMC10198973; doi:10.1038/s41598-023-35389-w)
Supplement: Supplementary file 1 — Supplementary Tables. [file 41598_2023_35389_MOESM1_ESM.docx]

**Table 1.** Results of Classification by Machine Learning Using 18F-flortaucipir-PET SUVR

| **Models** | **Classification** | **Variables** | **Accuracy** | **Precision** | **Recall** | **F1 Score** | **AUC** |
| --- | --- | --- | --- | --- | --- | --- | --- |
| **SVM** | **CU-AD** | Clinical | 0.96±0.00 | 0.92±0.00 | 1.00±0.00 | 0.96±0.00 | 1.00±0.01 |
|  |  | Tau | 0.92±0.00 | 0.91±0.00 | 0.91±0.00 | 0.91±0.00 | 0.94±0.01 |
|  |  | Clinical with Tau | 0.96±0.00 | 0.92±0.00 | 1.00±0.00 | 0.96±0.00 | 1.00±0.00 |
|  | **CU-MCI** | Clinical | 0.76±0.00 | 0.89±0.00 | 0.57±0.00 | 0.70±0.00 | 0.68±0.01 |
|  |  | Tau | 0.81±0.02 | 0.91±0.00 | 0.69±0.00 | 0.78±0.03 | 0.72±0.02 |
|  |  | Clinical with Tau | 0.81±0.02 | 0.82±0.03 | 0.79±0.04 | 0.80±0.02 | 0.80±0.02 |

The classification using machine learning. Clinical variables consist of age, sex, education, and MMSE. Tau variables mean tau SUVR for target region such as amygdala, entorhinal, fusiform, temporal and para-hippocampal.

Abbreviations: AUC; Area under the receiver operating characteristic curve, CU; cognitively unimpaired, MCI; mild cognitive impairment, AD; Alzheimer’s disease; PET, positron emission tomography, SUVR; Standard Uptake Value Ratio, SVM; Support Vector Machine.

Values are presented as mean ± SD unless otherwise stated.

**Table 2.** Subjects list for classification between cognitive um-impairment and Alzheimer’s disease.

|  | **Train** | | | | **Test** | | | |
| --- | --- | --- | --- | --- | --- | --- | --- | --- |
|  | **CU** | | **AD** | | **CU** | | **AD** | |
|  | **ID** | **Date** | **ID** | **Date** | **ID** | **Date** | **ID** | **Date** |
| **1** | 073_S_6925 | 20210526 | 003_S_6264 | 20181127 | 168_S_6492 | 20181018 | 135_S_7003 | 20211025 |
| **2** | 082_S_6283 | 20180618 | 003_S_6833 | 20200109 | 177_S_6328 | 20180607 | 153_S_6694 |  |
| **3** | 082_S_6287 | 20180718 | 006_S_6689 | 20190523 | 301_S_6224 | 20180308 | 168_S_6142 | 20180116 |
| **4** | 094_S_6417 | 20180724 | 011_S_6303 | 20180507 | 305_S_6157 | 20180315 | 168_S_6735 | 20191106 |
| **5** | 094_S_6419 | 20180725 | 012_S_6837 | 20200109 | 305_S_6188 | 20180329 | 168_S_6754 | 20191029 |
| **6** | 094_S_6468 | 20180829 | 013_S_6768 | 20191010 | 305_S_6313 | 20180509 | 168_S_6828 | 20191210 |
| **7** | 094_S_6485 | 20180808 | 016_S_6708 | 20190717 | 341_S_6494 | 20180920 | 168_S_6843 | 20200317 |
| **8** | 098_S_6343 | 20180821 | 016_S_6839 | 20200205 | 941_S_6054 | 20170801 | 301_S_6592 | 20181113 |
| **9** | 098_S_6734 | 20191216 | 019_S_6573 | 20180926 | 941_S_6384 | 20180606 | 305_S_6810 | 20210105 |
| **10** | 099_S_6016 | 20170824 | 022_S_6013 | 20170510 | 941_S_6454 | 20180724 | 305_S_6850 | 20200213 |
| **11** | 099_S_6175 | 20180410 | 022_S_6796 | 20190926 | 941_S_6499 | 20180731 | 341_S_6820 | 20191121 |
| **12** | 100_S_6349 | 20180529 | 022_S_6863 | 20200916 | 941_S_6570 | 20180926 | 941_S_6854 | 20200309 |
| **13** | 114_S_6429 | 20181205 | 023_S_6661 | 20190228 | 941_S_6575 | 20181024 | 941_S_6962 | 20210817 |
| **14** | 114_S_6487 | 20190227 | 027_S_6648 | 20190219 |  |  |  |  |
| **15** | 114_S_6524 | 20190306 | 027_S_6733 | 20190626 |  |  |  |  |
| **16** | 116_S_6439 | 20180821 | 027_S_6849 | 20200310 |  |  |  |  |
| **17** | 126_S_6559 | 20181010 | 027_S_6965 | 20210713 |  |  |  |  |
| **18** | 127_S_6348 | 20180808 | 032_S_6600 | 20181115 |  |  |  |  |
| **19** | 129_S_6146 | 20180117 | 032_S_6602 | 20181113 |  |  |  |  |
| **20** | 129_S_6244 | 20180328 | 033_S_6705 | 20190509 |  |  |  |  |
| **21** | 129_S_6459 | 20180711 | 033_S_6976 | 20210803 |  |  |  |  |
| **22** | 135_S_6359 | 20180625 | 035_S_6927 | 20210624 |  |  |  |  |
| **23** | 135_S_6473 | 20181016 | 036_S_6179 | 20180307 |  |  |  |  |
| **24** | 135_S_6509 | 20180809 | 036_S_6231 | 20180405 |  |  |  |  |
| **25** | 141_S_6008 | 20170530 | 037_S_6216 | 20180705 |  |  |  |  |
| **26** | 141_S_6116 | 20171128 | 037_S_6377 | 20180817 |  |  |  |  |
| **27** | 141_S_6240 | 20180327 | 057_S_6869 | 20210430 |  |  |  |  |
| **28** | 141_S_6416 | 20181101 | 098_S_6601 | 20181207 |  |  |  |  |
| **29** | 141_S_6423 | 20180829 | 098_S_6655 | 20190513 |  |  |  |  |
| **30** | 141_S_6589 | 20190103 | 098_S_6658 | 20190410 |  |  |  |  |
| **31** | 168_S_6049 | 20180508 | 100_S_6713 | 20190528 |  |  |  |  |
| **32** | 168_S_6085 | 20171212 | 114_S_6039 | 20171004 |  |  |  |  |
| **33** | 168_S_6151 | 20180412 | 114_S_6347 | 20181219 |  |  |  |  |
| **34** | 168_S_6318 | 20180606 | 114_S_6595 | 20190606 |  |  |  |  |
| **35** | 168_S_6320 | 20180531 | 116_S_6100 | 20180309 |  |  |  |  |
| **36** | 135_S_6360 | 20180606 | 116_S_6543 | 20180924 |  |  |  |  |
| **37** | 135_S_6411 | 20180619 | 123_S_6825 | 20200929 |  |  |  |  |
| **38** | 135_S_6510 | 20180827 | 123_S_6891 | 20201117 |  |  |  |  |
| **39** | 168_S_6233 | 20180613 | 126_S_6683 | 20190301 |  |  |  |  |
| **40** | 168_S_6281 | 20180801 | 126_S_6721 | 20190605 |  |  |  |  |
| **41** | 168_S_6285 | 20180607 | 127_S_6433 | 20180829 |  |  |  |  |
| **42** | 168_S_6321 | 20180613 | 127_S_6549 | 20181022 |  |  |  |  |
| **43** | 168_S_6350 | 20180718 | 129_S_6763 | 20190725 |  |  |  |  |
| **44** | 168_S_6371 | 20180920 | 129_S_6784 | 20190813 |  |  |  |  |
| **45** | 168_S_6413 | 20180809 | 130_S_6072 | 20170915 |  |  |  |  |
| **46** | 177_S_6335 | 20180626 | 135_S_6284 | 20180507 |  |  |  |  |
| **47** | 177_S_6408 | 20180709 | 135_S_6389 | 20180703 |  |  |  |  |
| **48** | 177_S_6409 | 20180703 | 135_S_6545 | 20180914 |  |  |  |  |
| **49** | 941_S_6471 | 20180724 | 135_S_6687 | 20190312 |  |  |  |  |
| **50** | 941_S_6581 | 20180921 | 135_S_6840 | 20191216 |  |  |  |  |

The list of subjects for this study acquired from ADNI. Abbreviations: CU, cognitive unimpaired; AD, Alzheimer’s Disease; ID, patient ID; Date, data acquisition date.

**Table 3.** Subjects list for classification between cognitive um-impairment and mild cognitive impairment.

|  | **Train** | | | | Test | | | |
| --- | --- | --- | --- | --- | --- | --- | --- | --- |
|  | **CU** | | **MCI** | | **CU** | | **MCI** | |
|  | **ID** | **Date** | **ID** | **Date** | **ID** | **Date** | **ID** | **Date** |
| **1** | 003_S_6924 | 20210429 | 003_S_6606 | 20190228 | 036_S_6466 | 20180904 | 127_S_6241 | 20180813 |
| **2** | 003_S_6014 | 20180410 | 006_S_6252 | 20180425 | 037_S_6144 | 20180425 | 127_S_6512 | 20190117 |
| **3** | 003_S_6067 | 20171018 | 003_S_6479 | 20181129 | 037_S_6187 | 20180621 | 129_S_6830 | 20191107 |
| **4** | 003_S_6256 | 20180705 | 002_S_6695 | 20190410 | 037_S_6204 | 20180502 | 130_S_6611 | 20190207 |
| **5** | 006_S_6277 | 20180507 | 010_S_6748 | 20190821 | 037_S_6956 | 20210915 | 130_S_6612 | 20190110 |
| **6** | 007_S_6521 | 20181115 | 007_S_6341 | 20180530 | 041_S_6136 | 20171214 | 130_S_6688 | 20190307 |
| **7** | 016_S_6381 | 20180531 | 006_S_6291 | 20180517 | 041_S_6292 | 20180426 | 131_S_6143 | 20180808 |
| **8** | 014_S_6145 | 20180315 | 009_S_6402 | 20180904 | 041_S_6354 | 20180531 | 131_S_6616 | 20190425 |
| **9** | 011_S_6465 | 20180808 | 006_S_6681 | 20190314 | 067_S_6045 | 20170822 | 135_S_6586 | 20181114 |
| **10** | 014_S_6199 | 20180322 | 006_S_6610 | 20181205 | 067_S_6443 | 20180926 | 135_S_6703 | 20190424 |
| **11** | 014_S_6437 | 20181010 | 006_S_6657 | 20190412 | 067_S_6528 | 20181107 | 137_S_6654 | 20190314 |
| **12** | 016_S_6802 | 20191211 | 006_S_6727 | 20190718 | 067_S_6958 | 20211014 | 137_S_6693 | 20191219 |
| **13** | 016_S_6773 | 20190911 | 006_S_6441 | 20180912 | 070_S_6394 | 20180912 | 141_S_6075 | 20171012 |
| **14** | 011_S_6367 | 20180531 | 011_S_6618 | 20190723 | 070_S_6542 | 20180918 | 141_S_6964 | 20210720 |
| **15** | 020_S_6504 | 20181203 | 016_S_6809 | 20191211 | 073_S_6907 | 20210511 | 153_S_6336 | 20180620 |
| **16** | 021_S_6918 | 20210407 | 019_S_6668 | 20190220 |  |  |  |  |
| **17** | 021_S_6940 | 20210525 | 019_S_6533 | 20180904 |  |  |  |  |
| **18** | 020_S_6185 | 20180410 | 019_S_6315 | 20180509 |  |  |  |  |
| **19** | 021_S_6910 | 20210318 | 012_S_6073 | 20171011 |  |  |  |  |
| **20** | 018_S_6207 | 20180228 | 016_S_6800 | 20191210 |  |  |  |  |
| **21** | 021_S_6896 | 20210121 | 019_S_6483 | 20180725 |  |  |  |  |
| **22** | 021_S_6914 | 20210408 | 019_S_6757 | 20190627 |  |  |  |  |
| **23** | 020_S_6566 | 20190220 | 014_S_6765 | 20191009 |  |  |  |  |
| **24** | 018_S_6351 | 20180523 | 023_S_6334 | 20180613 |  |  |  |  |
| **25** | 024_S_6202 | 20180323 | 027_S_6788 | 20190828 |  |  |  |  |
| **26** | 021_S_6987 | 20210810 | 027_S_6370 | 20180815 |  |  |  |  |
| **27** | 024_S_6472 | 20180801 | 027_S_6640 | 20190207 |  |  |  |  |
| **28** | 021_S_6994 | 20210908 | 032_S_6804 | 20191203 |  |  |  |  |
| **29** | 024_S_6005 | 20170427 | 032_S_6700 | 20191024 |  |  |  |  |
| **30** | 023_S_6346 | 20180809 | 023_S_6356 | 20180718 |  |  |  |  |
| **31** | 027_S_6577 | 20181107 | 023_S_6535 | 20181009 |  |  |  |  |
| **32** | 022_S_6797 | 20191002 | 027_S_6002 | 20170329 |  |  |  |  |
| **33** | 027_S_6183 | 20180222 | 027_S_7007 | 20211026 |  |  |  |  |
| **34** | 024_S_6385 | 20180627 | 027_S_6643 | 20190124 |  |  |  |  |
| **35** | 035_S_6551 | 20180823 | 036_S_6134 | 20180103 |  |  |  |  |
| **36** | 032_S_6709 | 20190611 | 036_S_6885 | 20201021 |  |  |  |  |
| **37** | 035_S_6488 | 20180816 | 036_S_6894 | 20201202 |  |  |  |  |
| **38** | 029_S_6289 | 20180426 | 036_S_6897 | 20210217 |  |  |  |  |
| **39** | 033_S_6352 | 20180618 | 036_S_6916 | 20210407 |  |  |  |  |
| **40** | 029_S_6505 | 20181009 | 036_S_6878 | 20200805 |  |  |  |  |
| **41** | 032_S_6701 | 20190910 | 037_S_6125 | 20180222 |  |  |  |  |
| **42** | 032_S_6293 | 20180725 | 035_S_7000 | 20211013 |  |  |  |  |
| **43** | 032_S_6211 | 20180410 | 035_S_6641 | 20190115 |  |  |  |  |
| **44** | 027_S_6582 | 20181018 | 033_S_6889 | 20201016 |  |  |  |  |
| **45** | 035_S_6950 | 20210715 | 070_S_6229 | 20180328 |  |  |  |  |
| **46** | 100_S_6493 | 20181004 | 051_S_6761 | 20190806 |  |  |  |  |
| **47** | 100_S_6578 | 20181009 | 070_S_6236 | 20180319 |  |  |  |  |
| **48** | 027_S_6001 | 20170327 | 098_S_6593 | 20190220 |  |  |  |  |
| **49** | 114_S_6057 | 20180405 | 067_S_6989 | 20211020 |  |  |  |  |
| **50** | 114_S_6113 | 20180621 | 037_S_6141 | 20180330 |  |  |  |  |
| **51** | 114_S_6251 | 20180711 | 067_S_6529 | 20181010 |  |  |  |  |
| **52** | 123_S_6118 | 20180726 | 052_S_6832 | 20200211 |  |  |  |  |
| **53** | 127_S_6232 | 20180521 | 070_S_6911 | 20210415 |  |  |  |  |
| **54** | 127_S_6330 | 20180614 | 099_S_6632 | 20190129 |  |  |  |  |
| **55** | 127_S_6357 | 20180829 | 116_S_6428 | 20180821 |  |  |  |  |
| **56** | 127_S_6436 | 20180808 | 126_S_6724 | 20190627 |  |  |  |  |
| **57** | 129_S_6452 | 20180726 | 116_S_6550 | 20180829 |  |  |  |  |
| **58** | 129_S_6457 | 20180726 | 109_S_6300 | 20190315 |  |  |  |  |
| **59** | 130_S_6319 | 20180613 | 114_S_6597 | 20190328 |  |  |  |  |
| **60** | 131_S_6170 | 20180808 | 109_S_6373 | 20190207 |  |  |  |  |

The list of subjects for this study acquired from ADNI. Abbreviations: CU, cognitive unimpaired; MCI, mild cognitive impairment; ID, patient ID; Date, data acquisition date.
